# OVERVIEW OF NIA RESEARCH

**DOI:** 10.1093/geroni/igz038.1507

**Published:** 2019-11-08

**Authors:** Richard Hodes

**Affiliations:** National Institute on Aging, National Institutes of Health, Bethesda, Maryland, United States

## Abstract

Dr. Hodes will provide an overview of the latest research in basic biology, neuroscience, behavioral and social science, and geriatrics and clinical gerontology supported by the NIA.

